# Methodology for radiochromic film analysis using FilmQA Pro and ImageJ

**DOI:** 10.1371/journal.pone.0233562

**Published:** 2020-05-21

**Authors:** Michelle E. Howard, Michael G. Herman, Michael P. Grams

**Affiliations:** Department of Radiation Oncology, Mayo Clinic, Rochester, Minnesota, United States of America; University of Seville, SPAIN

## Abstract

Radiochromic film (RCF) has several advantageous characteristics which make it an attractive dosimeter for many clinical tasks in radiation oncology. However, knowledge of and strict adherence to complicated protocols in order to produce accurate measurements can prohibit RCF from being widely adopted in the clinic. The purpose of this study was to outline some simple and straightforward RCF fundamentals in order to help clinical medical physicists perform accurate RCF measurements. We describe a process and methodology successfully used in our practice with the hope that it saves time and effort for others when implementing RCF in their clinics. Two RCF analysis software programs which differ in cost and complexity, the commercially available FilmQA Pro package and the freely available ImageJ software, were used to show the accuracy, consistency and limitations of each. The process described resulted in a majority of the measurements across a wide dose range to be accurate within ± 2% of the intended dose using either FilmQA Pro or ImageJ.

## Introduction

Identifying a single dosimeter suitable for a diverse range of clinical tasks in radiation oncology can be challenging. Radiochromic film (RCF) is an attractive option due to its relatively low cost, high spatial resolution, near tissue equivalence [1], dose rate independence [2], angular independence [3], temperature independence up to 60°C and energy independence over a large range of therapeutic MV energies [4–8]. Furthermore, RCF is flexible, waterproof, can be cut to various sizes, and provides two dimensional dose measurements. These attributes have proven useful for a variety of clinical tasks such as dosimetry for total skin electron therapy [9, 10] or total body irradiation [11]. RCF is also used in patient specific quality assurance (QA) measurements for delivery techniques such as volumetric modulated arc therapy (VMAT) [12] or stereotactic radiosurgery (SRS) [13, 14] where high spatial resolution is necessary and has proven useful as a research tool in phantom and radiobiological studies to verify dosimetry in unconventional geometries that do not easily accommodate conventional dosimeters [15–21].

Despite the many advantages of RCF, it has perhaps not gained widespread acceptance as an accurate and reliable dosimeter since its overall accuracy can depend on subtle, yet important factors. For example, a number of variables such as film flatness [22], the lateral response artifact [23, 24], scanner behavior [25–27] and general film handling can compromise measurements and increase uncertainty. These issues can be overcome with strict adherence to protocols, calibration procedures and scanning processes, but require awareness of the potential problems, their causes, and solutions. Therefore, establishing a reliable film dosimetry practice can include substantial effort and time by the user. A condensed and straightforward description outlining a reliable and consistent process to use RCF may save the clinical physicist time by avoiding mistakes which lead to frustrating and inconsistent results.

The aim of this work is to describe a process for handling, calibrating, scanning and analyzing film, which in our experience, has allowed for accurate and consistent dosimetric measurements across a wide range of clinically relevant doses. While there are many excellent papers [6, 20, 28–34] and an AAPM Task Group Report [35] which provide a vast amount of valuable information relevant to film dosimetry, the process outlined here is meant to be a concise summary of the necessary basic fundamentals that are required for accurate RCF dosimetry. The methods described have been used in our clinic for more than six years, both in routine clinical tasks such as in vivo TSE measurements [36, 37] and IMRT QA [14] as well as in research in conventional x-ray [16, 21, 38] and proton therapy [39] studies. While certainly not the only approach for successful film dosimetry, they should help potential RCF users to implement RCF into clinical practice within a relatively quick timeframe. Potential sources of error that may adversely affect the film measurements and how to avoid them are described. Further, this paper outlines the process of analyzing RCF using two software packages: FilmQA Pro (Ashland, Bridgewater, NJ, USA), an advanced commercially available package involving an expedited scanning protocol [40] with triple-channel dosimetry [41–43]; and ImageJ, a freely available open source image analysis platform with single channel dosimetry (NIH, Bethesda, MD, USA)[44]. There are many commercially available software packages available for RCF film analysis, and developing in-house software is also common. Use of the two software packages in this report should not be considered as an endorsement of either one, but rather were chosen to illustrate that the fundamentals described are equally applicable to advanced, commercially available software (FilmQA Pro) as well as freely available packages (ImageJ). Furthermore, the methods described are also applicable to in-house software packages.

## Methods

### Equipment used in this work

The basic equipment required is RCF, a guillotine paper cutter, a document scanner and analysis software. All measurements in this study utilized either Gafchromic EBT3 or EBT-XD film (Ashland, Bridgewater, NJ, USA) for doses ranging from 0–1000 cGy and 0–3000 cGy respectively. Precise cuts by a guillotine paper cutter were made to minimize damage to the edges of the film strips which can manifest by the individual layers of film separating. Alternatively, a sharp scissors may also be used. Scanning of the film was done using an Epson 10000 XL flatbed document scanner (version 3.49A) and analysis was done with both FilmQA Pro and ImageJ. RCF used to generate the calibration curves and test the accuracy of the calibration were from the same manufactured lot.

### Creation of a calibration curve

**Fig 1:**
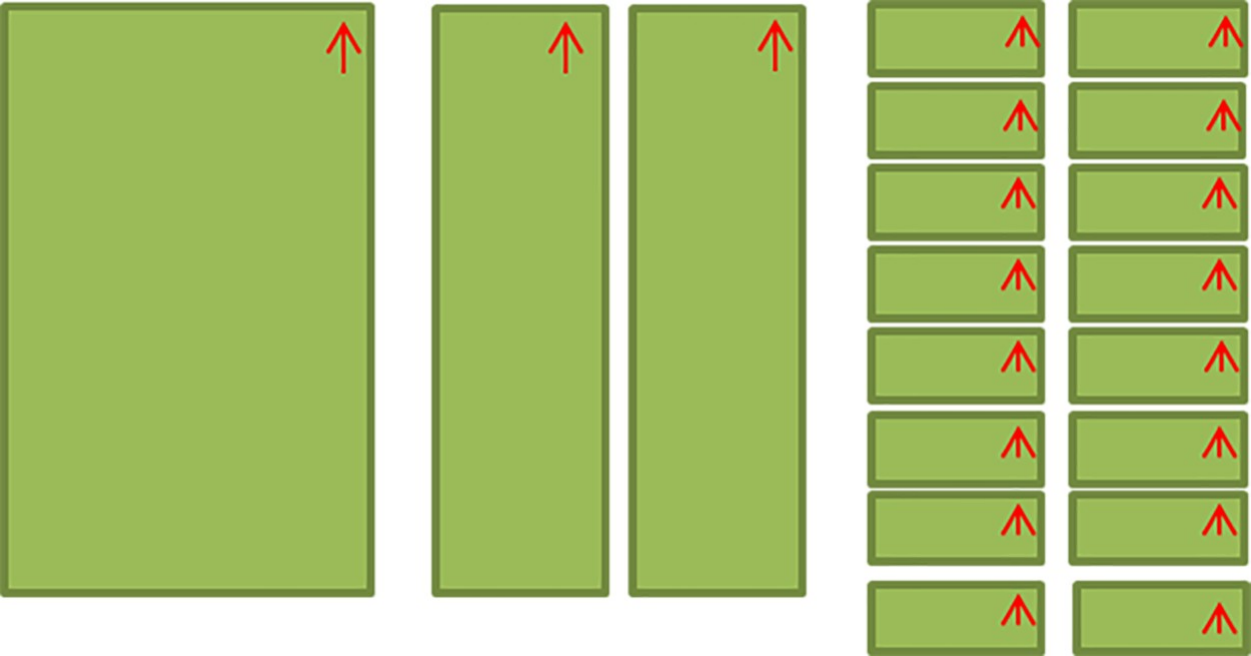
A diagram outlining the film cutting and orientation indicating process for a single sheet.

Calibration curves consisting of multiple dose points for various dose ranges were generated: 0–300 cGy with 4 dose points using EBT3; 0–1000 cGy with 7 dose points using EBT3; and 0–3000 cGy with 8 dose points using EBT-XD. Curves were generated within each software package. The films used for calibration were made by cutting a single sheet of RCF (20.32 x 25.4 cm^2^) into 16 smaller strips (approximately 2.54 x 12.7 cm^2^). Since there is known to be a directional dependence when scanning film with conventional document scanners [45], film orientation must be kept consistent during the cutting of each sheet so all films can eventually be scanned with the same orientation. Film orientation was indicated by placing an arrow in the top right corner of each strip, facing the same direction for the entire sheet of film. Fig 1 depicts the cutting of one sheet of film into 16 individual strips of the same size, with arrows in the top right corner, parallel to the longer (25.4 cm) side of the film, to ensure a consistent scanning orientation.

Prior to irradiating film strips used for the calibration curve, the output of the linear accelerator (Varian Truebeam) was verified using an ADCL calibrated ionization chamber. Film strips were then exposed one at a time to the desired calibration doses using 6 MV x-rays at the depth of maximum dose (1.3 cm) in solid water with 10 cm of backscatter. Other depths can be used for exposure of calibration films as long as the delivered dose at depth can be accurately verified. The plane of the film was placed perpendicular to the axis of the beam using a 10 x 10 cm^2^ field at 100 cm SSD. During exposure, the remaining film strips were kept outside of the linear accelerator vault and away from ambient light. Calibration films were scanned 24 hours after irradiation to allow for stabilization of post-irradiation growth of the active layer in the films.

**Fig 2:**
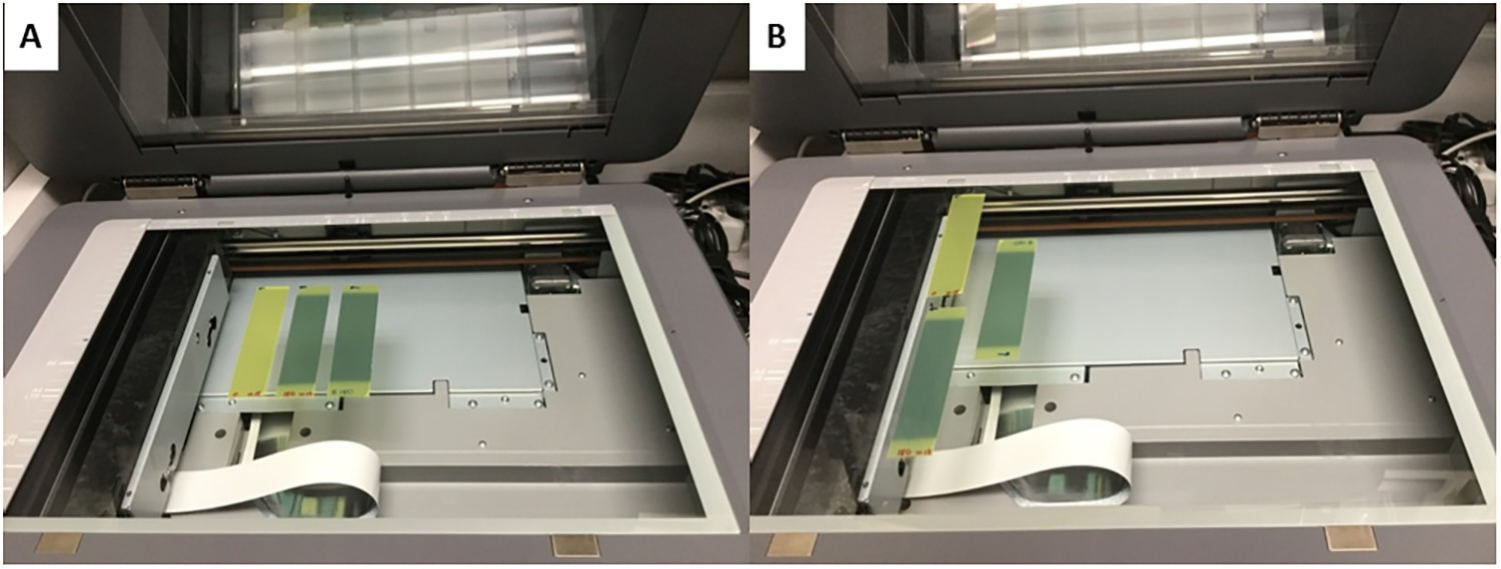
Orientation of radiochromic film. A) Film aligned centrally on the flatbed scanner. B) Film placed improperly on the flatbed scanner.

Film strips were placed in the center of the document scanner with the orientation arrow on the strips pointing in the same direction (Fig 2A). A glass plate was placed over the films on the scanner bed to ensure the films were flat for the duration of the scan. Films were scanned in red-green-blue (RGB) format using a 48-bit scanner at 72 dpi, in transmission mode, and with no color or sharpness corrections. Three warm up scans were conducted prior to the eventual scan used for calibration. Images of the calibration strips were saved in TIFF format and then opened for analysis in both FilmQA Pro and ImageJ for analysis.

#### Calibration curve generation using FilmQA Pro

**Fig 3:**
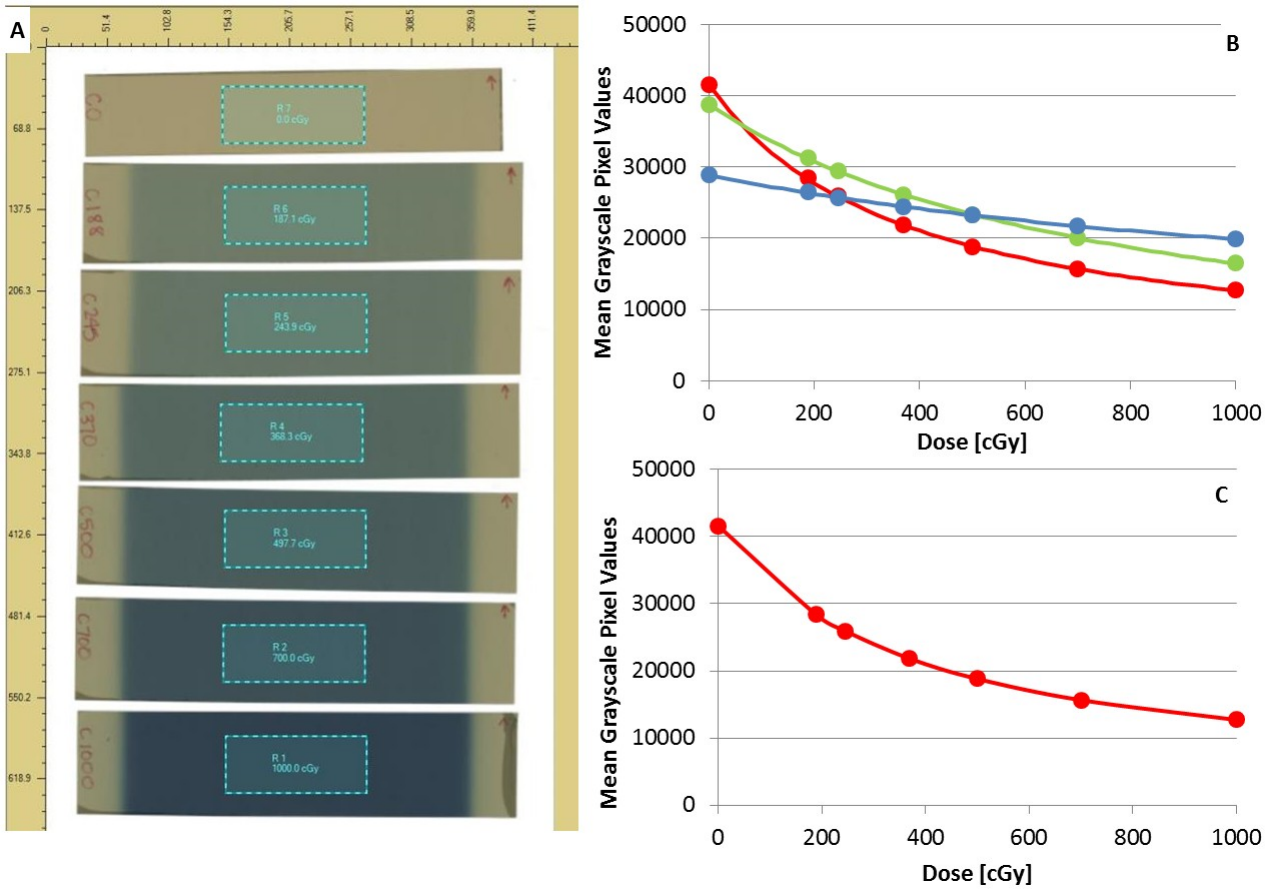
Radiochromic film processing and calibration procedure. A) ROI placement on film; B) Calibration curve made with FilmQA Pro; C) calibration curve made with ImageJ.

A region of interest (ROI), over which the response of the film is quantified, was created for each film strip. ROI’s of the same size (~2 x 4 cm^2^) were placed in the center of each calibration film and the delivered doses were entered into the software (Fig 3A). When employing triple channel dosimetry, discrete data points of film response and dose are fitted with a curve for the three separate color channels (Fig 3B). Various rational functions within the FilmQA Pro software are available to fit the film response [40]. The function selected to represent the calibration data is determined by visually inspecting the fit of the curve to the discrete data points as well as its performance in correctly determining known doses. FilmQA Pro provides quantitative information estimating the accuracy of the fit for each calibration function through relative consistency values. Consistency here refers to the ability of each RGB calibration function to return the same dose, i.e., to be “consistent” with each other. Consistency values for an accurate calibration are normally within 2–3 percent.

#### Calibration curve generation using ImageJ

ImageJ is able to open and analyze most common image file types such as JPEG and TIFF. The scanned in calibration film is automatically separated into each of the three color channels. While any of the three color channels can be used for dosimetry, our experience has shown the highest accuracy can be obtained up to 1000 cGy in EBT3 and up to 3000 cGy with EBT-XD film when using the red channel. Therefore, all analysis for this study was done using the red color channel data. Similar to the FilmQA Pro analysis protocol, an ROI was defined for each of the calibration films (Fig 3A). A mean grayscale value was measured for each film and entered into ImageJ in table format with the associated delivered doses. The relationship between mean grayscale values and dose can then be fit using a number of functions provided in the software (Fig 3C). The “Rodbard (NIH image)” function, defined in Eq 1, proved to accurately quantify measured dose when comparing films exposed to known doses across multiple functions.

$$y=c*{\left(\frac{x-a}{d-x}\right)}^{1/b}$$ (1)

The coefficients a, b, c and d in the equation above are defined within ImageJ based on the mean grayscale values, x, and expected doses input during calibration. Future film measurements can use this calibration function by measuring the mean grayscale values and substituting each into Eq 1 to solve for dose (y). This analysis can be scripted or calculated in a spreadsheet.

### Validation of the calibration curve

To test the accuracy of the calibration curves, additional strips of film from the same batch were cut and irradiated with known doses and then analyzed. These test films were exposed following the same setup conditions used for the calibration curves (6 MV x-rays at 1.3 cm depth in solid water with a 10 x 10 cm^2^ field at 100 cm SSD). The doses delivered to the test films covered the entire range of the calibration curve. Dose values were evenly spaced within each dose range and three films were exposed per point on three separate days (Table 1) in order to establish the consistency of the process and also to mimic a more clinically relevant scenario where measurements are needed on a routine basis. The size of the ROIs for all test films were kept consistent for both FilmQA Pro and ImageJ analysis: W = 110 pixels; H = 53 pixels; ~2 x 4 cm^2^.

**Table 1:** Test film information.

GafChromic Film Type	Dose Range [cGy]	Reference Film Doses [cGy]	Tested Dose Points [cGy]
EBT3	0–300	0, 270	0, 50, 100, 150, 200, 250, 300
EBT3	0–1000	0, 900	0, 100, 200, 300, 400, 500, 600, 700, 800, 900, 1000
EBTXD	0–3000	0, 2700	0, 300, 600, 900, 1200, 1500, 1800, 2100, 2400, 2700, 3000

#### Analysis of the test films using FilmQA Pro

The same scanning technique as outlined above was followed, using an expedited scanning protocol available within the FilmQA Pro software which is described in detail elsewhere [40]. Briefly, the so called “single scan protocol” requires simultaneously scanning the film for which the dose is unknown along with two “reference” films: one irradiated at a known dose within approximately ±10% of the maximum expected measured dose and one unexposed film. The reference films provide data by which the calibration function can be rescaled within the software to compensate for differences in time between exposure and scanning of the films to be analyzed and the time that was allowed between exposures and scanning of the calibration films. In the single scan protocol, the reference film should be irradiated as soon as possible before or after other film measurements are completed. If all films are exposed within a narrow time window, the film analysis can be completed in as little as 20 minutes rather than having to wait 24 hours or more as is common when using other RCF protocols. Reference films were exposed either immediately before or after test films, since the order of exposure does not impact the accuracy of results. Each batch of test strips was analyzed with reference films at 0 cGy and either 270, 900, or 2700 cGy, depending on the maximum dose of each group of films.

Triple channel dosimetry [41] was employed for all analysis using FilmQA Pro. In triple channel dosimetry, the average dose response from the calibration curves of the red, green and blue color channels are used to separate dose dependent contributions to film response from non-dose dependent disturbances due to non-uniformity in the active layer of the film, finger prints, or noise within the scanner readout system. As the absorbed dose to the film must be independent of color channel, triple channel methods are designed to find disturbance values which minimize the difference in dose between the separate color channels. Once this disturbance value is determined, it can be used to remove or minimize the dose-independent response contributions. Triple channel methods have been shown to improve the overall accuracy of RCF dosimetry [46, 47].

After scanning the test films and application of the single scan protocol, the mean dose for each film was sampled over an approximately 2 x 4 cm^2^ area centered on the exposed region and compared against the known delivered dose. The same sized ROIs were used for each test film to measure dose.

#### Analysis of the test films using ImageJ

The same films from the FilmQA Pro analysis were also used for ImageJ analysis by saving a TIFF file of the scanned image. Therefore, the time between exposure and scanning of 24 hours was kept constant for the test strips and matched the timing of the calibration films. The single color channel technique, the most basic methodology for RCF dosimetry, was utilized for ImageJ analysis. This technique converts any measured signal to a dose response, leaving the measurement susceptible to artifacts such as fingerprints or non-uniformities in the active layer that may ultimately translate into dosimetric errors.

Using the red channel, ROIs of the same size, as specified above, were used to take measurements of mean grayscale values for each film. The ROI dimensions were specified to match those used in the calibration curves and test film analysis for both FilmQA Pro and ImageJ. The measured mean gray scale values served as the x-value input data to the calibration function (Eq 1), where the y-value output is given in dose (cGy). For ImageJ analysis, the reference strips were not used as ImageJ does not support a single scan protocol similar to FilmQA Pro. Consequently, users of ImageJ must wait the same amount of time between irradiation and scanning of application films as they waited between exposing and scanning the calibration films.

### Data analysis for the comparison FilmQA Pro and ImageJ

In order to assess the accuracy of both film analysis tools (FilmQA Pro and ImageJ) used in this study, a comparison between measured and intended dose was plotted for both. Error bars represent ± two standard deviations from the mean of the dose sampled within the ROI on each film. Further, the percent difference from the intended dose was plotted to investigate the accuracy of each analysis tool. Finally, a Bland-Altman plot or difference plot was used to assess the agreement between two measurement tools. Bland-Altman plots are also able to identify any systematic differences between the measurements to determine if a fixed bias is present.

## Results

**Fig 4:**
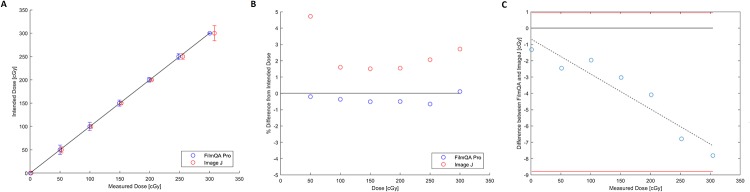
EBT3 for the 0–300 cGy dose range. A) Measured vs. intended dose. The solid line has a slope of one and is meant to guide the eye, as points falling on it indicate perfect agreement. Error bars represent 3 standard deviations. B) Percent difference from intended dose. C) Bland-Altman Plot.

**Fig 5:**
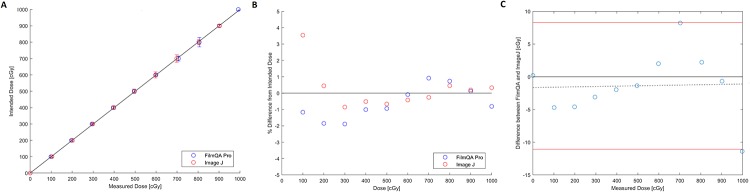
EBT3 for the 0–1000 cGy dose range. A) Measured vs. intended dose. The solid line has a slope of one and is meant to guide the eye, as points falling on it indicate perfect agreement. Error bars represent 3 standard deviations. B) Percent difference from intended dose. C) Bland-Altman Plot.

**Fig 6:**
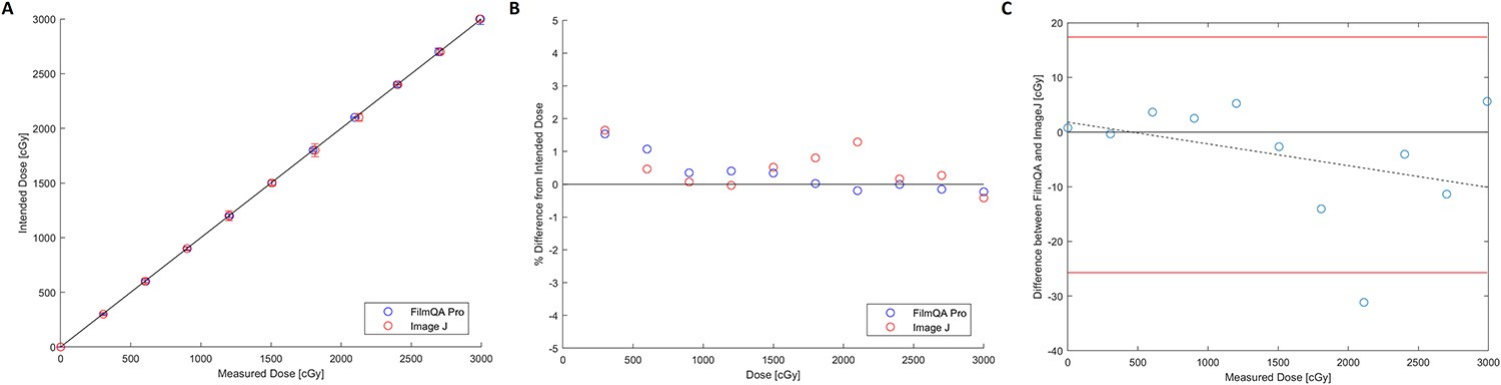
EBTXD for the 0–3000 cGy dose range. A) Measured vs. intended dose. The solid line has a slope of one and is meant to guide the eye, as points falling on it indicate perfect agreement. Error bars represent 3 standard deviations. B) Percent difference from intended dose. C) Bland-Altman Plot.

The average dose from three independent measurements was compared to the expected doses. Film analyses using FilmQA Pro and ImageJ both resulted in dosimetric measurements within ± 5% of the intended dose delivered with a majority of the results within ± 2% (Figs 4–6). Measurements differing from expected values by 3–5% were at low doses of 100 cGy or less where signal to noise ratios become challenging for RCF analysis. Though the percentage errors at doses below 100 cGy may seem large, for an expected dose of 40 cGy a measurement of 42 cGy represents a 5% error but may be considered clinically insignificant in terms of absolute dose.

For the EBT3 measurements of doses ranging from 0–300 cGy, measurements were all within ± 1% of expected values using FilmQA Pro and within ± 4.7% of expected values for ImageJ. All ImageJ results from the 0–300 cGy dose range measured higher than the intended dose, with the largest discrepancy of 4.7% (2.4 cGy) between measured and intended dose being at the lowest dose point of 50 cGy (Fig 4B). The Bland-Altman plot showed the difference in measurements between FilmQA Pro and ImageJ, where the steep slope of the mean difference shows the two methods of analysis are not well correlated (Fig 4C). This is made apparent when considering the data points for the two analyses methods in Fig 4A are distinctively spread apart at higher doses. A line with a slope of one was included only to guide the eye and to show instances where measured data points are in perfect agreement with the intended dose.

Film results from the 0–1000 cGy dose range using EBT3 show good agreement for both FilmQA Pro and ImageJ between the average across three independent measurements and intended dose delivered (Fig 5A & 5B). All but one mean measured value were within ± 2% of their respective expected doses, as indicated by all data points lying on or next to the line with a slope of one. The outlier present in the ImageJ analysis was the measurement at 100 cGy, the lowest dose point in the range of tested doses, similar to results from the 0–300 cGy data (Fig 5B). Overall, dose measurements were similar from FilmQA Pro and ImageJ, as shown in the Bland-Altman plot, where the mean difference line was flat (Fig 5C).

Finally, EBTXD measurements ranging from 0–3000 cGy showed good agreement with the intended dose to within ± 1% where the majority of measurements were higher than the expected doses (Fig 6A & 6B). Both FilmQA Pro and ImageJ produced comparable dose readings, as illustrated in the shallow slope of the mean difference line on the Bland-Altman plot (Fig 6C).

## Discussion and conclusions

A strict protocol should be followed when using RCF as an absolute dosimeter to ensure accurate and reproducible results. The protocol described in this paper uses the following basic concepts:

- Cut film with a guillotine cutter, or otherwise suitably sharp edge, to minimize damage to the film. Mark each piece of film cut from the same sheet so a consistent orientation on the scanner can always be maintained.
- Irradiate films used for calibration with a setup in which the delivered dose is well defined.
- Film placement in the center of the scanner, in a single row along the scan direction to avoid the influence of the lateral scan effect. Use a glass plate to keep the films flat on the scanner bed.
- A new calibration curve should be established and tested for each unique lot of RCF. Assess the accuracy of the calibration curve and analysis process by irradiating additional films to known doses throughout the range of the calibration and comparing expected and measured values. Single measurements are sufficient for verification of the calibration curve once a protocol is established for clinical use.

When used consistently within the parameters of an appropriately established protocol, RCF can be an accurate and clinically useful dosimeter. This work provides a relatively simple outline for film handling and analysis that resulted in accurate and consistent dosimetric measurements across a wide range of clinically relevant doses. Additionally, two analysis tools, FilmQA Pro and ImageJ, were shown to provide comparably accurate results for the studied dose ranges. It should be emphasized that the methods and fundamentals summarized at the beginning of this section are independent of the software used for RCF analysis, and are equally applicable to other commercial software packages as well as those developed in-house.

While the commercially available package FilmQA Pro has the advantage of an expedited scanning protocol and triple channel dosimetry, accurate single channel dosimetry with the freely available ImageJ software is also possible. Depending on the desired accuracy level and the application for which RCF is to be used, the purchase of a commercial software package may not be necessary. For example, simple dosimetric measurements might be carried out sufficiently with ImageJ but the analysis of patient specific IMRT QA measurements might be better suited for a more sophisticated commercial package. One main difference between the two software packages is the use of single vs. triple channel dosimetry. FilmQA Pro allows either single or triple channel options for analysis, and triple channel methods have been shown to generally be more accurate. ImageJ is limited to the single channel technique which likely contributes to the differences shown in the results where FilmQA Pro tends to provide better accuracy.

While in this work multiple calibration curves were created covering different dose ranges, users of radiochromic film may choose to create a single calibration curve which encompasses the expected dose range needed for all clinical dosimetry tasks. Martin-Viera Cueto et al. [48] have described universal calibration curves which are valid over wide dose ranges. These universal calibrations are valid since their parameters are related to the physical mechanism associated with film exposure, namely, the activation of the crystalline polydiacetylene molecules in the active layer. Similarly, Casolara et al. [49, 50] have shown parallels between the responses of radiochromic and photographic film and therefore the ability to describe the dynamic developing process of both through a general equation to create a universal calibration curve. As these universal calibration curves are described by parameters which depend on the response of the sensitive material in the film, and most films share the same active material, they are broadly applicable across various types of film. The methods and fundamentals described in this paper for creating and testing the accuracy of any type of universal calibration are still valid, and should be employed prior to its clinical use.

Results from this study showed the ability of RCF to produce accurate dose measurements to within ± 2% of the expected dose for a majority of the tested doses, regardless of the software used. However, the accuracy of measurements taken at doses less than 100 cGy is likely to be lower, where individual measurements deviated from expected values up to 5.5% and 7.4% for 50–100 cGy and <50 cGy, respectively. Measurements made in the 50–100 cGy range are still useful as these uncertainties translate to differences of a few cGy. For example, the individual measured values for 100 cGy ranged from 96.6–102.3 cGy and 100.3–105.5 cGy for FilmQA Pro and ImageJ, respectively.

A versatile radiation dosimeter that is accurate, rugged, flexible, waterproof and largely independent of temperature, energy and dose rate can be very useful within the clinic for a variety of applications. We have demonstrated the accuracy of the RCF protocol outlined above across a wide range of clinical doses. Further, the resulting dosimetric measurements were similar across two data analysis software packages for the majority of measurements. Therefore, analysis software required for dependable measurements is largely dependent on the level of accuracy required for the associated task. Through careful development and adherence to protocol, RCF can be a dependable absolute dosimeter for a variety of tasks both clinically and in research.

## References

[1] Williams M, Metcalfe P. Radiochromic Film Dosimetry and its Applications in Radiotherapy2011.

[2] Karsch L, Beyreuther E, Burris-Mog T, Kraft S, Richter C, Zeil K, et al Dose rate dependence for different dosimeters and detectors: TLD, OSL, EBT films, and diamond detectors. Medical physics. 2012;39(5):2447–55. Epub 2012/05/09. 10.1118/1.3700400 . 22559615

[3] Kairn T, Hardcastle N, Kenny J, Meldrum R, Tome WA, Aland T. EBT2 radiochromic film for quality assurance of complex IMRT treatments of the prostate: micro-collimated IMRT, RapidArc, and TomoTherapy. Australasian physical & engineering sciences in medicine. 2011;34(3):333–43. Epub 2011/07/13. 10.1007/s13246-011-0087-z . 21748444

[4] Massillon-Jl G, Chiu-Tsao S-T, Muñoz I, Chan M. Energy Dependence of the New Gafchromic EBT3 Film: Dose Response Curves for 50 kV, 6 and 15 MV X-Ray Beams. International Journal of Medical Physics, Clinical Engineering and Radiation Oncology. 2012;1:60–5. 10.4236/ijmpcero.2012.12008

[5] Cheung T, Butson MJ, Yu PK. Independence of calibration curves for EBT Gafchromic films of the size of high-energy X-ray fields. Applied radiation and isotopes: including data, instrumentation and methods for use in agriculture, industry and medicine. 2006;64(9):1027–30. Epub 2006/06/16. 10.1016/j.apradiso.2006.04.006 . 16774834

[6] Fuss M, Sturtewagen E, De Wagter C, Georg D. Dosimetric characterization of GafChromic EBT film and its implication on film dosimetry quality assurance. Physics in medicine and biology. 2007;52(14):4211–25. Epub 2007/08/01. 10.1088/0031-9155/52/14/013 . 17664604

[7] Rink A, Vitkin IA, Jaffray DA. Energy dependence (75 kVp to 18 MV) of radiochromic films assessed using a real-time optical dosimeter. Medical physics. 2007;34(2):458–63. Epub 2007/03/29. 10.1118/1.2431425 . 17388161

[8] Sorriaux J, Kacperek A, Rossomme S, Lee JA, Bertrand D, Vynckier S, et al Evaluation of Gafchromic(R) EBT3 films characteristics in therapy photon, electron and proton beams. Physica medica: PM: an international journal devoted to the applications of physics to medicine and biology: official journal of the Italian Association of Biomedical Physics (AIFB). 2013;29(6):599–606. Epub 2012/10/31. 10.1016/j.ejmp.2012.10.001 23107430

[9] Deufel CL, Antolak JA. Total skin electron therapy in the lying-on-the-floor position using a customized flattening filter to eliminate field junctions. Journal of applied clinical medical physics. 2013;14(5):115–26. Epub 2013/09/17. . 24036864 10.1120/jacmp.v14i5.4309 PMC5714577

[10] Falahati L, Nedaie HA, Esfahani M, Banaee N. Dosimetric evaluation of electron total skin irradiation using gafchromic film and thermoluminescent dosimetry. Journal of cancer research and therapeutics. 2019;15(Supplement):S115–s22. Epub 2019/03/23. 10.4103/jcrt.JCRT_1020_16 . 30900632

[11] Su FC, Shi C, Papanikolaou N. Clinical application of GAFCHROMIC EBT film for in vivo dose measurements of total body irradiation radiotherapy. Applied radiation and isotopes: including data, instrumentation and methods for use in agriculture, industry and medicine. 2008;66(3):389–94. Epub 2007/11/21. 10.1016/j.apradiso.2007.09.015 . 18023587

[12] Barbeiro AR, Ureba A, Baeza JA, Linares R, Perucha M, Jimenez-Ortega E, et al 3D VMAT Verification Based on Monte Carlo Log File Simulation with Experimental Feedback from Film Dosimetry. PLoS One. 2016;11(11):e0166767 Epub 2016/11/22. 10.1371/journal.pone.0166767 . 27870878 PMC5117721

[13] Brezovich IA, Wu X, Popple RA, Covington E, Cardan R, Shen S, et al Stereotactic radiosurgery with MLC-defined arcs: Verification of dosimetry, spatial accuracy, and end-to-end tests. Journal of applied clinical medical physics. 2019 Epub 2019/04/13. 10.1002/acm2.12583 . 30977297 PMC6522994

[14] Grams MP, de Los Santos LEF. Design and clinical use of a rotational phantom for dosimetric verification of IMRT/VMAT treatments. Physica medica: PM: an international journal devoted to the applications of physics to medicine and biology: official journal of the Italian Association of Biomedical Physics (AIFB). 2018;50:59–65. Epub 2018/06/13. 10.1016/j.ejmp.2018.05.019 . 29891095

[15] Howard M, Beltran C, Sarkaria J, Herman MG. Characterization of relative biological effectiveness for conventional radiation therapy: a comparison of clinical 6 MV X-rays and 137 Cs. Journal of radiation research. 2017:1–6. 10.1093/jrr/rrx018 . 28444207 PMC5737853

[16] Shiraishi S, Fong de Los Santos LE, Antolak JA, Olivier KR, Garces YI, Park SS, et al Phantom Verification of AAA and Acuros Dose Calculations for Lung Cancer: Do Tumor Size and Regression Matter? Practical radiation oncology. 2019;9(1):29–37. Epub 2018/08/24. 10.1016/j.prro.2018.06.008 . 30138746

[17] Rutherford A, Stevenson K, Tulk A, Chalmers AJ. Evaluation of four different small animal radiation plans on tumour and normal tissue dosimetry in a glioblastoma mouse model. The British journal of radiology. 2019;92(1095):20180469 Epub 2018/10/27. 10.1259/bjr.20180469 . 30362815 PMC6541187

[18] Ghita M, McMahon SJ, Thompson HF, McGarry CK, King R, Osman SOS, et al Small field dosimetry for the small animal radiotherapy research platform (SARRP). Radiation oncology (London, England). 2017;12(1):204 Epub 2017/12/29. 10.1186/s13014-017-0936-3 . 29282134 PMC5745702

[19] Girard F, Bouchard H, Lacroix F. Reference dosimetry using radiochromic film. Journal of applied clinical medical physics. 2012;13(6):3994 Epub 2012/11/15. 10.1120/jacmp.v13i6.3994 . 23149793 PMC5718535

[20] Bouchard H, Lacroix F, Beaudoin G, Carrier JF, Kawrakow I. On the characterization and uncertainty analysis of radiochromic film dosimetry. Medical physics. 2009;36(6):1931–46. Epub 2009/07/21. 10.1118/1.3121488 . 19610282

[21] Grams MP, Fong de Los Santos LE, Antolak JA, Brinkmann DH, Clarke MJ, Park SS, et al Cadaveric verification of the Eclipse AAA algorithm for spine SBRT treatments with titanium hardware. Practical radiation oncology. 2016;6(2):131–41. Epub 2016/01/03. 10.1016/j.prro.2015.10.012 . 26723553

[22] Palmer AL, Bradley DA, Nisbet A. Evaluation and mitigation of potential errors in radiochromic film dosimetry due to film curvature at scanning. Journal of applied clinical medical physics. 2015;16(2):5141 Epub 2015/06/24. 10.1120/jacmp.v16i2.5141 . 26103181 PMC5690100

[23] Lewis D, Chan MF. Correcting lateral response artifacts from flatbed scanners for radiochromic film dosimetry. Medical physics. 2015;42(1):416–29. Epub 2015/01/08. 10.1118/1.4903758 . 25563282 PMC5148133

[24] Lewis DF, Chan MF. Technical Note: On GAFChromic EBT-XD film and the lateral response artifact. Medical physics. 2016;43(2):643–9. Epub 2016/02/05. 10.1118/1.4939226 . 26843228 PMC4715006

[25] Matney JE, Parker BC, Neck DW, Henkelmann G, Rosen II. Evaluation of a commercial flatbed document scanner and radiographic film scanner for radiochromic EBT film dosimetry. Journal of applied clinical medical physics. 2010;11(2):3165-. 10.1120/jacmp.v11i2.3165 . 20592699 PMC5719943

[26] Tagiling N, Ab Rashid R, Azhan SNA, Dollah N, Geso M, Rahman WN. Effect of scanning parameters on dose-response of radiochromic films irradiated with photon and electron beams. Heliyon. 2018;4(10):e00864–e. 10.1016/j.heliyon.2018.e00864 . 30364574 PMC6197593

[27] Alnawaf H, Yu PKN, Butson M. Comparison of Epson scanner quality for radiochromic film evaluation. Journal of applied clinical medical physics. 2012;13(5):3957-. 10.1120/jacmp.v13i5.3957 . 22955661 PMC5718226

[28] Devic S, Tomic N, Lewis D. Reference radiochromic film dosimetry: Review of technical aspects. Physica medica: PM: an international journal devoted to the applications of physics to medicine and biology: official journal of the Italian Association of Biomedical Physics (AIFB). 2016;32(4):541–56. Epub 2016/03/30. 10.1016/j.ejmp.2016.02.008 . 27020097

[29] Soares CG. Radiochromic film dosimetry. Radiation Measurements. 2006;41:S100–S16. 10.1016/j.radmeas.2007.01.007. 16987914

[30] Schoenfeld AA, Poppinga D, Harder D, Doerner KJ, Poppe B. The artefacts of radiochromic film dosimetry with flatbed scanners and their causation by light scattering from radiation-induced polymers. Physics in medicine and biology. 2014;59(13):3575–97. Epub 2014/06/10. 10.1088/0031-9155/59/13/3575 . 24909235

[31] van Battum LJ, Huizenga H, Verdaasdonk RM, Heukelom S. How flatbed scanners upset accurate film dosimetry. Physics in medicine and biology. 2016;61(2):625–49. Epub 2015/12/23. 10.1088/0031-9155/61/2/625 . 26689962

[32] Lynch BD, Kozelka J, Ranade MK, Li JG, Simon WE, Dempsey JF. Important considerations for radiochromic film dosimetry with flatbed CCD scanners and EBT GAFCHROMIC film. Medical physics. 2006;33(12):4551–6. Epub 2007/02/07. 10.1118/1.2370505 . 17278806

[33] Devic S, Seuntjens J, Sham E, Podgorsak EB, Schmidtlein CR, Kirov AS, et al Precise radiochromic film dosimetry using a flat-bed document scanner. Medical physics. 2005;32(7):2245–53. Epub 2005/08/27. 10.1118/1.1929253 . 16121579

[34] Devic S. Radiochromic film dosimetry: past, present, and future. Physica medica: PM: an international journal devoted to the applications of physics to medicine and biology: official journal of the Italian Association of Biomedical Physics (AIFB). 2011;27(3):122–34. Epub 2010/11/06. 10.1016/j.ejmp.2010.10.001 . 21050785

[35] Niroomand-Rad A, Blackwell CR, Coursey BM, Gall KP, Galvin JM, McLaughlin WL, et al Radiochromic film dosimetry: recommendations of AAPM Radiation Therapy Committee Task Group 55. American Association of Physicists in Medicine. Medical physics. 1998;25(11):2093–115. Epub 1998/11/26. 10.1118/1.598407 . 9829234

[36] Evans JD, Haley LL, Locher SE, Grams MP, Deufel CL, Antolak JA, et al Clinical application of lying-on-the-floor total skin electron irradiation for frail patients with cutaneous lymphoma: An emphasis on the importance of in vivo dosimetry. Adv Radiat Oncol. 2016;1(2):101–5. 10.1016/j.adro.2016.03.005 . 28740876 PMC5506731

[37] Ahmed SK, Grams MP, Locher SE, McLemore LB, Sio TT, Martenson JA. Adaptation of the Stanford technique for treatment of bulky cutaneous T-cell lymphoma of the head. Practical radiation oncology. 2016;6(3):183–6. Epub 2015/12/30. 10.1016/j.prro.2015.10.021 . 26712465

[38] Howard M, Beltran C, Sarkaria J, Herman MG. Characterization of relative biological effectiveness for conventional radiation therapy: a comparison of clinical 6 MV X-rays and 137 Cs. Journal of radiation research. 2017;58(5):608–13. Epub 2017/04/27. 10.1093/jrr/rrx018 . 28444207 PMC5737853

[39] Anderson SE, Grams MP, Wan Chan Tseung H, Furutani KM, Beltran CJ. A linear relationship for the LET-dependence of Gafchromic EBT3 film in spot-scanning proton therapy. Physics in medicine and biology. 2019;64(5):055015 Epub 2019/01/24. 10.1088/1361-6560/ab0114 . 30673655

[40] Lewis D, Micke A, Yu X, Chan MF. An efficient protocol for radiochromic film dosimetry combining calibration and measurement in a single scan. Medical physics. 2012;39(10):6339–50. Epub 2012/10/09. 10.1118/1.4754797 . 23039670 PMC9381144

[41] Micke A, Lewis DF, Yu X. Multichannel film dosimetry with nonuniformity correction. Medical physics. 2011;38(5):2523–34. Epub 2011/07/23. 10.1118/1.3576105 . 21776787

[42] Perez Azorin JF, Ramos Garcia LI, Marti-Climent JM. A method for multichannel dosimetry with EBT3 radiochromic films. Medical physics. 2014;41(6):062101 Epub 2014/06/01. 10.1118/1.4871622 . 24877828

[43] Mendez I, Peterlin P, Hudej R, Strojnik A, Casar B. On multichannel film dosimetry with channel-independent perturbations. Medical physics. 2014;41(1):011705 Epub 2014/01/07. 10.1118/1.4845095 . 24387497

[44] Abramoff M, Magalhães P, Ram SJ. Image Processing with ImageJ. Biophotonics International. 2003;11:36–42.

[45] Dreindl R, Georg D, Stock M. Radiochromic film dosimetry: considerations on precision and accuracy for EBT2 and EBT3 type films. Zeitschrift fur medizinische Physik. 2014;24(2):153–63. Epub 2013/09/24. 10.1016/j.zemedi.2013.08.002 . 24055395

[46] Palmer AL, Bradley D, Nisbet A. Evaluation and implementation of triple-channel radiochromic film dosimetry in brachytherapy. Journal of applied clinical medical physics. 2014;15(4):4854-. 10.1120/jacmp.v15i4.4854 . 25207417 PMC5875501

[47] van Hoof SJ, Granton PV, Landry G, Podesta M, Verhaegen F. Evaluation of a novel triple-channel radiochromic film analysis procedure using EBT2. Physics in medicine and biology. 2012;57(13):4353–68. Epub 2012/06/19. 10.1088/0031-9155/57/13/4353 . 22705890

[48] Martin-Viera Cueto JA, Parra Osorio V, Moreno Saiz C, Navarro Guirado F, Casado Villalon FJ, Galan Montenegro P. A universal dose-response curve for radiochromic films. Medical physics. 2015;42(1):221–31. Epub 2015/01/08. 10.1118/1.4903301 . 25563262

[49] Casolaro P, Campajola L, Capua FD. The physics of radiochromic process: one calibration equation for all film types. Journal of Instrumentation. 2019;14(08):P08006–P. 10.1088/1748-0221/14/08/p08006

[50] Campajola L, Casolaro P, Capua FD. Absolute dose calibration of EBT3 Gafchromic films. Journal of Instrumentation. 2017;12(08):P08015–P. 10.1088/1748-0221/12/08/p08015

